# Supplementary material for: Is active sitting on a dynamic office chair controlled by the trunk muscles?
Source: PLoS One. 2020 Nov 30;15(11):e0242854. doi: 10.1371/journal.pone.0242854 (PMC7703901; doi:10.1371/journal.pone.0242854)
Supplement: Attachment — Submitted filename: Response to Reviewers.docx [file pone.0242854.s001.docx]

Dear Editor, Dear Reviewers

We highly appreciate and thank for the critical input on our manuscript formerly entitled *Active sitting on a dynamic office chair – Is it trunk muscle controlled?* helping us to improve its quality. Please find below a point-to-point answer to all comments. Due to the major changes in the manuscript, the submitted version with track changes is hard to read, and we decided to refer in our answers to the clean version of the manuscript.

Please note that we changed the manuscript title to *“Is active sitting on a dynamic office chair controlled by the trunk muscles?”*

Reviewer #1

Major comments
1. Although the authors state that they consulted assistance for writing the manuscript, the manuscript contains several grammatical errors and some sentences are difficult to understand. The manuscript should be carefully copy-edited prior to resubmission.

The language of the manuscript was professionally revised before resubmission. As a consequence, the manuscript title changed to *“Is active sitting on a dynamic office chair controlled by the trunk muscles?”*

2. The study design is not fully suitable for answering the study objective as the mechanism of controlling sitting on the dynamic chair cannot be experimentally elucidated. Nonetheless, observed difference in study parameters between conditions are highly relevant. The conclusions should be revised accordingly.

Following your detailed comments, we revised the conclusions accordingly.

3. The authors postulate that there are three different mechanisms for achieving seat motion. Why is it either or? It is also possible that it is a combination of these mechanisms. How can you unlink these mechanisms? Trunk muscle activity is necessary to initiate trunk motion.

We agree that the motion could be a result of a combination of these mechanisms. We present the study objectives accordingly, and argue in the discussion why the trunk muscle activity pattern is likely the main reason for the seat motion (and not the consequence of upper body motions as the upper body remains very stable and in upright position): *Due to the stable upper body posture, the observed trunk muscle activity pattern is likely to have been the dominant factor controlling the seat movement. The trunk muscles are not needed to stabilise the upper body, because the upper body remains stable and upright during active sitting. (*Line 320-323)

4. The authors state in their second objective that the study quantifies active sitting on the new chair regarding muscular activity and body segment kinematics, and analyses the immediate effect of a spinal RoM training on muscular activity and body segment kinematics. This is the main aim of the study. The first objective is a mere interpretation of these results and would be appropriate in the discussion section. To proof these interrelationships would necessitate a musculoskeletal model where each of the stated factors is modulated systematically.

We agree with the reviewer that the second aim was our primary aim and the primary one is its interpretation. We revised the manuscript accordingly (see Line 86-92)

5. The authors may consider restructuring their manuscript. For instance, shouldn’t the outcome parameter be the result of the data processing? Please rearrange the methods section to follow a logical flow.

The method section was restructured following the detailed comments.

6. The authors repeatedly mention spinal ROM training yet miss to describe what they mean. Moreover, this study did not involve any training and hence all statements regarding training should be removed.

As this study did not involve any training, all statements regarding the ROM training were removed.

Minor Comments

**ABSTRACT**
1. Abstract: The objective should be rephrased as specified in the comments to the introduction section.

Objective rephrased accordingly: *The study aimed to determine the means by which the seat movement was controlled during active sitting. This was accomplished by quantifying the muscular trunk activity, thigh activity and body kinematics. Additionally, the effect of increased spinal motion on muscular activity and body kinematics was analysed.* (Line 20-23)

2. Abstract: The conclusion is not supported by the data presented in this study. See also comments in the introduction and discussion sections.

Conclusion rephrased accordingly: *The characteristic activity pattern of the lumbar trunk muscles was shown to be the most likely dominant factor in controlling seat movement during active sitting. Consequently, the new chair could have a potential positive impact on back health during prolonged sitting. Further studies are necessary to analyse the frequency and intensity of active sitting during daily office work.* (Line 36-40)

**INTRODUCTION**
3. Line 43: to develop -> of developing

Revised as recommended, line 43.

4. Line 45: colons are followed by lower case. While -> while. Please check throughout the manuscript as this mistake is repeated several times in the text.

Colon removed, line 45.

5. Line 47: to develop -> of developing

Revised as recommended, line 48.

6. Line 49: Better: This may lead to deconditioning of lumbar spine muscles?

… *which could lead to deconditioning of the lumbar spine* by the language reviewer, line 49-59.

7. Line 49: LBP patients. Not defining patients by their disease is more respectful of the person. Please change to patients with LBP throughout the manuscript.

Revised as recommended in the entire manuscript.

8. Line 65: having only -> with only

Revised as recommended, line 68.

9. Line 66: in order to -> to

Revised to *for maintaining balance* by the language reviewer, line 68-69.

10. Line 67 and entire manuscript. Please carefully review the literature - for instance, this paper is missing: Effects of a Dynamic Chair on Chair Seat Motion and Trunk Muscle Activity during Office Tasks and Task Transitions.

We thank the reviewer for this suggestion, and included the mentioned reference in the introduction (Ref 24, line 65)*.*

11. Line 76-77: “…that the upper body remains during active sitting within a range considered to be stable” -> “…that during active sitting the upper body remains within a range considered to be stable”

Revised to *the upper body remained within a range considered to be stable during active sitting* by the language reviewer (line 81).

12. Line 81: it was so far not known -> to date it is unknown

Revised to *is still unknown* by the language reviewer (line 85-86).

13. Line 82 and entire manuscript: carefully check the tense. The purpose statement should be in past tense.

Carefully checked and revised in the entire manuscript with the help of the language reviewer.

14. Line 82: to uncover -> better: to determine

In line with major comment 4 revised to *to quantify* by the language reviewer (line 86)

15. Line 82-84: Why is it either or? It is also possible that it is a combination of these mechanisms. How can you unlink these mechanisms? Trunk muscle activity is necessary to initiate trunk motion.

The reviewer is right that it is not either or. Accordingly, we revised the section to *If muscular activity contributes to the control of the seat movement…* and *If body weight shift contributes to the control of the seat movement …* line 87-89

16. Line 83: latter – this is not the correct term. The intended meaning is unclear.

Sentence completely revised, see answer to major comment 4

17. Line 92: Opposed to instable chairs -> In contrast to other instable chairs

Revised to “*In contrast to instable chairs*” (line 94-95). The investigated office chair is not an instable one, see answer to next comment

18. Line 93: without muscular effort – without a user? What is your intended meaning?

Revised to … *the seat has a stable central position that rests at the lowest point of the arc corresponding to the centre of the seat.* lLine 95-96

**MATERIALS AND METHODS**

19. Line 97: Participants of this study…. -> This study was part of a larger study on….

Revised accordingly to: *This study was part of a larger study on active sitting and the recordings were made during the second session, described in Kuster et al. 2016* (line 99-100)

20. Line 98: The – delete

Revised accordingly

21. Line 99: Please include inclusion and exclusion criteria for the participants of this study.

Inclusion and exclusion criteria included: *Inclusion criteria were: 1) Age between 20 to 50 years; 2) Height between 1.53m and 1.92m; 3) BMI <30; 4) Working hours of >4 hours per day in a seated position over ≥2 years. Exclusion criteria were: 1) Chronic complaints of the back and neck region in the past year; 2) Previous spine surgery.* Line 101-105*.*

22. Line 100: Please provide SI units -> m

Revised accordingly, line 107.

23. Line 100: body weight -> body mass

Revised accordingly, line 107.

24. Line 101: better: … of which 6.5 +/- 2.1 hours were spent in a seated posture

Revised accordingly, line 108.

25. Line 102: Better: None of the participants had any experience with the investigated office chair prior to the study.

Revised to *None of the participants had prior experience of the investigated office chair prior to the study* by the language reviewer, line 109-110.

26. Line 103: by institutional -> by the institutional

Revised accordingly, line 110.

27. Line 106: Please provide more information on sample size calculation.

There was no sample size calculation: *Since no preliminary data were available, a convenient sample of n=10 was included based on information from similar studies.* (Line 112-113)

28. Line 110: The additional degree…. – phrase differently: slide side to side on a radius with a centre…

Revised accordingly, line 117-118.

29. Line 114: better: … because it will rest at the lowest point of the arc corresponding to the centre of the seat…

Revised to *the seat maintained a stable central position because it rested at the lowest point of the arc corresponding to the centre of the seat* by the language reviewer (line 121-122)

30. Line 115: better: …no additional muscle activity should be needed to…

Revised to *no additional muscle activity was needed to keep the seat position centred* by the language reviewer (line 123)

31. Line 116. Split sentence: The same chair…

Revised accordingly, line 123.

32. Line 118: start with: Muscle activity was recorded using…

Revised accordingly, line 151. (Section moved backwards due to major comment 5)

33. Line 121: Please include citation for the seniam guidelines.

Reference included, line 154.

34. Lines 122ff: There is no reason for abbreviating muscle names. These abbreviations make the text more difficult to read and should be removed throughout the manuscript. Moreover, please use lower case for all muscle names consistently throughout the manuscript, e.g. multifidus muscle….

Abbreviations removed in the entire manuscript.

35. Line 124: start with: Body kinematics were recorded using…

Revised accordingly, line 157.

36. Line 126: reflective body markers -> reflective skin markers

Revised accordingly, line 159

37. Line 127: Please provide information on marker size and reference to the PIG model.

Reference to PIG model (line 161) and marker size included (line 159).

38. Table 1: Please define all abbreviations in the table footnotes.

Abbreviations included in footnote of Table 1.

39. Line 132: on spine -> on the spine

Revised accordingly, line 164.

40. Line 134: additionally -> also

Revised accordingly, line 166

41. Line 140: was -> were

Revised accordingly

42. Line 141: to left -> to the left (did you only analyse seat motion from left to right?)

Revised accordingly, line 203. (Section moved backwards due to major comment 5)

*The movement cycle started and ended when the seat was in the central position and included the seat movements to the left and right side. Thus the movements to both sides were analysed.* Line 202-204

43. Line 143: average muscle activity: here, data processing was not explained yet. The average EMG signal will be close to 0. I assume you full-wave rectified the data and then computed the average? Please make sure that this is explained in the right order.

The outcome measure section (line 197-204) now follows the data processing section (line 168-196).

44. Line 146: The authors may consider moving this section to right after the chair description.

Moved as recommended, line 125-144.

45. Line 150: one minute -> 1 minute (numbers with units time always as digits)

Revised accordingly, line 130.

46. Line 151: better: Participants did not receive any movement instructions.

Revised to *without receiving movement instructions* by the language reviewer (line 130-131)

47. Line 154: Muscular -> sEMG

Revised accordingly, line 134.

48. Line 54: static sitting condition: Was the chair fixed in this position? Please clearly concisely the experimental conditions.

Statement included: *With the seat of the office chair fixed in the middle position* (line 134)

49. Line 168: How do you know that this is the optimal motion frequency?

We don’t know whether this is the “optimal” motion frequency. We noticed that subjects sitting for some weeks on the chair move the seat faster than subjects sitting for the first time on the chair. But we have not analysed an “optimal” frequency.

50. Line 161: When was this done? Before or after the seating conditions? Please specify.

*following the measurement of the sitting conditions* (line 142)

51. Line 165: Lateral and back view on… -> Lateral and posterior view of…

Revised accordingly, line 146.

52. Line 167: extremal seat positions -> maximum left and right positions

Revised accordingly, line 148.

53. Line 176: why did you use median and not mean?

*The median was taken because data were not normally distributed (verified with Lilliefors test),* line 175-176.

54. Line 177: data was -> data were (data is the plural of datum)

Revised accordingly, line 176

55. Line 181: statistical significance? This procedure is not clear. One should always choose clinical relevance over statistical significance.

To compare the temporal activity profile, we inspected the fraction of time the muscular activity is the same than for STATSIT, and the fraction of time it is higher and lower than for STATSIT. Accordingly, we removed the word “significance” as we have not performed a hypothesis testing, but only looked at the 95% range of STATSIT: *To compare the temporal activity profiles of ACTSIT and ACTSIT_max_ with STATSIT, the time in which the sEMG amplitude exceeded, was equal to, or fell below the 95% range of STATSIT was analysed and expressed as a percentage of the time.* Line 179-182.

56. Line 183: body segment thorax -> thorax segment

Revised accordingly, line 183.

57. Line 184: two maximum positions (to left and right) -> its maximum left and right positions

Revised accordingly, line 184.

58. Lien 184: halved -> divided by two

Revised accordingly, line 184.

59. Line 185: Was the movement symmetrical to both sides or did some subjects move more to one side than the other? This may influence muscle activity. The subject in Figure 2 has more spine curvature to the left than the right. It seems that this subject is not placed in the centre of the seat. This could largely influence the data. How did you control for this?

We asked the subjects to sit in the middle of the seat, but we have not measured whether they actually sat in the middle. We therefore checked whether the seat movement was symmetrical, and found no systematic differences.

Visually, it is true that the subject in figure 2 seems to have a larger spine curvature to the left. However, 1) this is just one example (…*show examples of…, line 147*), 2) the maximum positions for the figures were visually detected (from the seat wheels one can see that the motion to both sides is the same), 3) the spine markers do not form a straight line in the middle position. In conclusion, the larger spine curvature to the left might be just an optical issue.

60. Line 191: How were the spinal segments defined? Please specify.

*…angular difference between the uppermost (C7 to T4) and lowermost (L_low_ to SACR) spinal segments…*. Line 190-191, see also reference to Table 1, Line 189.

61. Line 199: against normality -> for normality (Please also specify the test you used to test for normality)

Revised to: *Data normality was tested with Lilliefors test* (line 206-207)

62. Lines 201ff: The statistical approach only addresses the second objective. How did you address the first objective?

See change in the objectives according to major comment 4.

**RESULTS**

63. Line 210: Figure 3 was unreadable – and hence I cannot comment on the data presented here.

We submitted all figure as recommended by the journal. However, to ensure the reviewer is able to see it, we additionally included Figure 3 in the end of the manuscript, right before reference list.

64. Line 229: Please do not use abbreviated muscle names as stated above.

Revised accordingly.

65. Line 230: significantly higher ILIO activity – compared to what?

*compared to STATSIT* inserted, line 242.

66. Line 232: Please provide statistical results of the post-hoc tests, ideally as 95% confidence interval of the difference.

Median with 95% Confidence Interval included in the result tables 2 and 4 as well as the statistics section of the methods: … *and significant effects were additionally described with a 95% confidence interval of the effect* (line 215-216).

67. Table 2: How do you explain the large variability in the data presented in this table?

See section about the sample size in the discussion: *Although the sample size is similar to that of previous studies in this field of research (20, 35), it limits the generalisability of the observed results and may partially explain the quite large variation observed in the presented results*. Line 383-385*.* And in the discussion about the reference value: *However, the absolute values presented in Table 2 would have been lower if a reference with higher activity was taken (e.g. maximum voluntary contraction).* Line 413-415.

68. Line 241: MULT and ILIO were…. – please rephrase this and the subsequent sentence as the intended meaning is not clear.

Revised to: *The muscle activity of multifidus and iliocostalis were higher during active sitting compared to static sitting for ≥49% of the time*. (line 255-257)

69. Line 248: Did the difference in left and right muscles occur during the same part of the activity? Why is the 50% mark important? Please show muscle activity patterns. Greater muscle activities in periods of greater muscle activity would be functionally more relevant.

The muscle activity pattern is plotted in Figure 3. The muscles on the left and right body side showed an opposite activation behaviour, see figure 3. The 50% were highlighted as this means that the muscle were most of the time on this activity level. However, we removed it. We cannot comment on the statement regarding the greater muscle activities because the alternative (STATSIT) has a constant muscular activity level.

70. Line 253: data in Table 4 is -> data in Table 4 are

Revised accordingly, line 268.

71. Table 4: Pleas report the 95% confidence interval of the difference.

Difference indicated with median and 95% confidence interval (Table 4).

**DISCUSSION**

72. Line 264: What about a combination of these mechanisms? See also comment to the introduction section.

We agree that the mechanisms can be used in combination, and revised the section accordingly. It now says that: *three mechanisms could be used to control the movement of the seat: a periodic activation of the trunk muscles; a periodic activation of the thigh muscles; and/or a substantial lateral shift in body weight.* Line 282-284*.*

73. Line 266: better: In particular, ILIO (don’t use abbreviation) was increased for both active sitting conditions compared to…

Revised accordingly, line 287-288.

74. Lines 268-273: Please do not repeat results in the discussion section. It is ok to summarize the results at a very high level.

We removed the results, and discuss the findings on a general level.

75. Line 277: preferred -> more efficient?

Revised accordingly, line 298.

76. Line 282: by a vice versa activity pattern -> by the opposite pattern?

Revised to *by the reverse pattern* by the language reviewer, line 303-304.

77. Line 283: How do you know that it is not the result of the motion? Additional activity might be necessary to stabilize the trunk when it is off centre.

*Due to the stable upper body posture, the observed trunk muscle activity pattern is likely to have been the dominant factor controlling the seat movement. The trunk muscles are not needed to stabilise the upper body, because the upper body remains stable and upright during active sitting.* Line 319-322*.*

78. Line 284: This may be because the thigh muscle activity is much lower during sitting than during walking. Again, here one would need to see the muscle activity patterns because a very small impulse might be sufficient for initiating the motion.

The statistical significance is independent of the chosen reference, and thus we would have observed the same results when taking another posture or activity as reference: *It must be noted that these reference values had no effect on the statistical analysis conducted in this study.* Line 412-413.

We noticed such a small impulse for the maximum active sitting condition, see Fig 3 and discussion: *…* *the results of the temporal analysis in Fig 3 show a slight variation in the thigh muscle activity level for ACTSIT_max_. This indicates that participants supported the trunk muscles with the thigh muscles to some degree to increase the RoM.* Line 306-309*.*

79. Line 288: One would need to look at the timing of the activity. Unfortunately, Fig 3 is not readable, so it is unclear if this statement is supported by the data.

Figure 3 is included in the manuscript before the references to ensure it is readable.

80. Line 293: How large is the variability between subjects? Did these patterns look the same for all subjects? Again, seeing Fig. 3 would be helpful.

The individual data is not included in Figure 3. The patterns look the same for all, otherwise the pattern would disappear in Figure 3.

81. Line 295: preceding -> precede

Revised accordingly, line 314

82. Line 296: post-hoc regression analysis -> correlation analysis (should also be explained in the statistics section)

We cancelled this section as we removed the spinal ROM training completely from the manuscript.

83. Line 297: Please include scatter plots showing data point for both sides and all subjects.

We cancelled this section as we removed the spinal ROM training completely from the manuscript.

84. Line 301: “…this observation is not an indication that the seat motion is controlled…” – Why not?

We cancelled this section as we removed the spinal ROM training completely from the manuscript. The answer is given in Comment 77: There was no substantial upper body motion.

85. Line 304: What is spinal ROM training?

We cancelled this section as we removed the spinal ROM training completely from the manuscript.

86. Line 307: By… -. When…

Revised accordingly, line 324.

87. Line 307: we notice -> we noticed

Revised to *was observed.* by the language reviewer, line 325.

88. Line 311: Again, here one would need to see the activation patterns to get an impression of the timing of greater and smaller activity.

See figure 3 at the end

89. Line 328: Was this the case for all subjects?

We are not sure what the reviewer means. If he/she means the speed of the lateral flexion in sitting and walking: We compared the speed of lateral flexion while active sitting to literature data for walking as we don’t have the data for walking recorded.

90. Line 330: Why now stair walking when you compare your values to treadmill walking?

As the motion speed was clearly above walking, we looked for another daily activity in a similar speed, which is double step stair ascent. Sentence revised to: *A similar movement speed can be found in double step stair ascen*t… Line 347-348.

91. Line 339: This sentence is incomplete.

Sentence removed. It does not add new information to the section.

92. Lines 340ff: This section does not relate to the study goals. The authors should focus on discussing the results of their study.

The centre of mass path and speed have been included in the results section (Table 4) and are discussed subsequently in the discussion section (Line 362)

93. Line 342: The authors did not report centre of mass speed in the results section. Please do not report additional results in the discussion section.

The centre of mass path and speed is reported in Table 4.

94. Line 345: does -> do

Revised to *Even the difference in methods, kinetic in Grooten et al. 2013 (27) versus kinematic (this study), does not explain* by the language reviewer (line 367-368).

95. Line 355: Please define what spinal range of motion training is. Here, the authors report acute differences in the outcome parameters yet lack to evaluate the efficacy of such training. Maybe patients with LBP would not even be able to complete these tasks?

The reviewer is right that we should be more critical about this topic. We therefore decided to remove this section from the manuscript.

96. Line 361: This speculation goes far beyond the objective of this study. Patients with LBP often have fatty atrophy that may be caused by neurological changes rather than by changes in physical activity.

We removed the section about spinal ROM training, and do not present any LBP causes.

97. Line 363: Again, one would need to consider the timing of these periods of increased and reduced muscle activity.

See Fig 3.

98. Line 374: Please rephrase this sentence.

Sentence removed.

99. Line 381: What would such training look like?

Following your comments, we removed the spinal ROM training completely.

100. Line 420: One limitation is that only ten subjects were enrolled in this study. Large variability in some results suggest limited generalisability of the results:

Statement included in the Critical appraisal*: Although the sample size is similar to that of previous studies in this field of research (20, 35), it limits the generalisability of the observed results and may partially explain the quite large variation observed in the presented results.* Line 383-385.

**CONCLUSION**

101. Lines 422ff: Again, it is likely a combination of these. To conclusively answer this question, one would need to implement a musculoskeletal model where the different parameters are altered systematically.

We agree with the reviewer that it does not has to be an either or question and revised it throughout the entire manuscript. However, the presented results strongly indicate that the trunk muscle activity is the main factor to control the seat motion. A detailed reasoning can be found in the second section of the discussion section (Line 285-322).

102. Line 427: This sentence does not relate to the results of this study.

Sentence removed.

Reviewer #2

Minor issues/comments:
- How long did the participants on average sit on the “active chair” during testing?

We have not measured the time they sat actively. Participants were asked to sit actively, and a total of 3 successive recordings, each with 6 motion cycles to both sides, were taken. Given that the files had to be saved in between, we assume that they sat for approximately 2 minutes active for the recordings.

- Have subjective responses/comments been assessed, on how sitting on this chair feels? Or whether participants would assume that sitting in this instable situation might be suitable for longer office working tasks?

Most participants commented on that, but the data were not collected in a structured way due to the short sitting time. However, most participants liked the motion, and asked whether they can use the chair for a day or so in their office.

- While the authors thoroughly discuss their results and carefully state the implications of their findings, it might be recommended to highlight that the actual benefits of this newly developed chair will need to be further tested under situations resembling more adequately real life circumstances (e.g. prolonged sitting for several hours, arm movements, changes from sitting to standing, turning while sitting,...). Furthermore its value will need to be assessed within the target population of people with low back pain.

We comment on that in the discussion section, line 335-338: *However, the intensity and frequency required to cause an effect for lumbar back muscles, as well as the feasibility of implementation during daily office work, remains a subject for future investigations* and in line 353-356: *a future study should analyse whether patients with LBP, whose back pain is relieved by walking, would also benefit from active sitting. If so, the chair might allow patients with LBP to get pain relief without interrupting their office work.*

We also comment on that in the conclusion section, line 439-440: *A future study should further investigate the use of the chair in a real office setting under normal working conditions.*

Miscellaneous remarks:
- Line 185: a typo?: …express the RoM of an unidirectional seat motion...

According to the language reviewer, it should be *a unidirectional… (*see also <https://www.a-or-an.com/a_an/unidirectional>)

- Figure caption 3: it is recommended to indicate that the data shown is “exemplary/typical data of one of the participants”

The data shown in Fig 3 are the average over all subjects, now clearly indicated: *Time normalized muscular activity and kinematic motion pattern during spontaneous (ACTSIT) and maximum active sitting (ACTSIT_max_) over all subjects*, line 233-235.
