# Supplementary material for: Is active sitting on a dynamic office chair controlled by the trunk muscles?
Source: PLoS One. 2020 Nov 30;15(11):e0242854. doi: 10.1371/journal.pone.0242854 (PMC7703901; doi:10.1371/journal.pone.0242854)
Supplement: Attachment — Submitted filename: Response to Reviewers.docx [file pone.0242854.s002.docx]

Dear Editor, Dear Reviewers

We highly appreciate and thank for the critical input on our manuscript entitled “*Is active sitting on a dynamic office chair controlled by the trunk muscles?”* helping us to improve its quality. Please find below a point-to-point answer to all comments. The indicated line numbers refer to the manuscript version with track changes.

Reviewer #1

General Comments
I would like to congratulate the authors for addressing all points adequately resulting in a very clear revised manuscript. The language has much improved, however there are still some awkward sentences especially in the introduction section that require attention.

We would like take the opportunity and thank the reviewer for the very detailed feedback we received. It was extremely helpful to improve the quality of the manuscript.

Minor Comments

1) Line 18: delete “, which was”

Revised as recommended, line 18.

2) Line 19: muscle-controlled -> muscle controlled

Revised as recommended, line 19.

3) Line 21: This was accomplished by quantifying the muscular trunk activity, thigh activity and body kinematics. -> This was accomplished by quantifying trunk and thigh muscular activity and body kinematics.

Revised as recommended, line 21-22.

4) Line 38: could -> may

Revised as recommended, line 38.

5) Line 48: It has been proposed that sitting-related LBP might be… -> Sitting-related LBP have been proposed to be…

Revised as recommended (has instead of have), line 48-49.

6) Line 55: for the health of the spine – Please be more specific

Revised to: “…*for the intervertebral discs and spinal muscles…*” , line 55-56.

7) Line 56: variations in -> altered

Revised as recommended, line 57.

8) Line 59: some 30% to 85% -> 30 to 85%

Revised as recommended, line 60.

9) Line 60: 34-90% -> 34 to 90%

Revised as recommended, line 61.

10) Line 62: made great efforts to promote -> promoted

Revised as recommended, line 62.

11) Line 68: (Fig 1), with only a small support base, to… – delete commata

Revised as recommended, line 68.

12) Lines 102- 105: Please use lower case for all points in the list.

Revised as recommended, line 102-105.

13) Lines 106-109: Delete “average” because mean is specified in brackets.

Revised as recommended, line 106-107.

14) Line 109: of the -> with the

Revised as recommended, line 109.

15) Line 100: delete “prior to the study”

Revised as recommended, line 110.

16) Line 129: sEMG -> (sEMG) – then use only abbreviation throughout the remainder of the manuscript

Revised as recommended, line 129 and 151.

17) Line 268ff: multifidus and iliocostalis between -> multifidus and iliocostalis muscular activity between – please also add muscular activity to all subsequent mentions of individual muscles.

Revised as recommended, line 287-292.

18) Line 310: If the shift in lateral body weight controls… -> If the lateral body shift controls…

Revised as recommended, line 311.

19) Line 339: How does active sitting affect the trunk kinematics? – Is this a subheading? If so, please correct the formatting.

Yes, it is, and we revised it as recommended, line 340.

Reviewer #2

Minor Comment

1) Line 102: inclusion criteria for height between 1.53 and 1.92 cm. Was this an inclusion criterion or it just happened to be the range of the respective participants?

This was actually the inclusion criteria to limit the study to heights in between the 5th percentile woman and 95th percentile man.
